# Impact of prenatal pyrethroid pesticide exposure on neonatal lymphocyte subsets and apoptosis: a birth cohort study

**DOI:** 10.7189/jogh.16.04318

**Published:** 2026-10-19

**Authors:** Zhiye Qi, Lili Zhang, Xia Xiao, Kun Wu, Shuqi Chen, Xiaoxiao Song, Yan Li

**Affiliations:** 1 Department of Pediatrics, First Affiliated Hospital of Kunming Medical University, Kunming, China; 2 School of Public Health, Kunming Medical University, Kunming, China; 3 Department of Pediatrics, Second Affiliated Hospital of Kunming Medical University, Kunming, China; 4 Department of Clinical Laboratory, First Affiliated Hospital of Kunming Medical University, Kunming, China; 5 Yunnan Key Laboratory of Laboratory Medicine, Kunming, China; 6 Haiyuan College, Kunming Medical University, Kunming, China

**Keywords:** pregnancy, pyrethroid, newborn, cellular immunity, apoptosis

## Abstract

**Background:**

Prenatal pyrethroid pesticides (PYRs) exposure may elevate infection diseases susceptibility of children. However, it is unclear the relationship of PYRs exposure and neonatal immune function. We explored the association of PYRs exposure during three trimesters with neonatal immunity and the roles of lymphocyte apoptosis.

**Methods:**

Maternal urinary PYRs metabolites (3PBA, 4F3PBA, *cis*-DBCA) were measured in each trimester. Cord blood lymphocyte subsets and lymphocyte apoptosis rates were assessed by flow cytometry. Multivariate linear regression models were used to examine the associations of trimester-specific and repeated PYRs exposure during pregnancy with neonatal immune function.

**Results:**

High-level *cis*-DBCA exposure (>75th percentile, 0.78 μg/g) in the third trimester was negatively associated with cord blood CD3^+^ T cell counts (β = −0.06; 95% CI = −0.12, −0.01) and CD8^+^ T cell counts (β = −0.09; 95% CI = −0.16, −0.01). Repeated high-level *cis*-DBCA exposure across two or more trimesters was associated with reduced CD3^+^ T cell counts (β = −0.09; 95% CI = −0.16, −0.02), CD4^+^ T cell counts (β = −0.13; 95% CI = −0.22, −0.04), CD8^+^ T cell counts (β = −0.13; 95% CI = −0.22, −0.04), B cell counts (β = −0.10; 95% CI = −0.20, −0.01), and NK cell counts (β = −0.14; 95% CI = −0.25, −0.03). High-level 3PBA exposure in the first trimester (β = 0.11; 95% CI = 0.01, 0.22) and high-level *cis*-DBCA exposure in the second trimester (β = 0.16; 95% CI = 0.04, 0.28) were positively associated with cord blood lymphocyte apoptosis rates. Repeated high-level *cis*-DBCA exposure in two or more trimesters was associated with increased cord blood lymphocyte apoptosis rates (β = 0.19; 95% CI = 0.03, 0.35).

**Conclusions:**

High-level maternal PYRs exposure during pregnancy may reduce neonatal lymphocyte counts and increase lymphocyte apoptosis. These effects persist throughout gestation, with the third trimester may represent a sensitive window for adverse effects on immune development.

The extensive use of pesticides has raised growing concerns about their contribution to environmental pollution and risks to human health [1], particularly their impact on child health [2]. Pyrethroid pesticides (PYRs) are characterised by high efficacy, low mammalian toxicity, and rapid degradation in the body, leading to their widespread application in crop pest control and household insecticides [3]. Once absorbed, PYRs are rapidly metabolised in the liver, and the resulting metabolites are excreted *via* the kidneys [4]. However, reduction of PYRs residues does not equate to complete elimination, as the metabolites may exhibit greater toxicity than the parent compounds [5]. The primary metabolites of PYRs include 3-phenoxybenzoic acid (3PBA), 4-fluoro-3-phenoxybenzoic acid (4F3PBA) and *cis*-3-(2,2-dibromovinyl)-2,2-dimethylcyclopropane carboxylic acid (*cis*-DBCA) [6]. Among these, 3PBA is a common metabolite of many PYRs, while *cis*-DBCA and 4F3PBA are the specific metabolites of deltamethrin and cyfluthrin, respectively [6].

Studies have shown that over 95% of pregnant women have been detected at least one PYRs metabolite during pregnancy [7,8]. Due to their high lipophilicity, PYRs can cross the placental barrier, leading to fetal exposure in utero [7]. The fetal period is characterised by rapid growth and functional maturation of tissues and organs, making it a highly sensitive window for environmental toxicants [9]. Consequently, the impact of prenatal PYRs exposure on offspring health has garnered significant attention.

Immune function plays a vital role in maintaining human health, and early-life represents a critical window for its development [10]. Exposure to hazardous substances during this period can lead to disruptions in immune system development, a condition termed Developmental Immunotoxicity (DIT) [11]. Exposure to harmful environmental factors at different stages of immune development may lead to various types of immune impairments [10]. Moreover, the developing immune system is more susceptible to immunotoxic damage induced by environmental exposures than the adult immune system [12], and such exposures may potentially lead to long-term health consequences for children [13]. Animal experiment has demonstrated that early-life exposure to PYRs alters immune cells and immune molecules [14] in offspring, thereby contributing to DIT. A cross-sectional study by Neta G *et al.* [15] reported an inverse correlation between cord blood PYRs metabolites and the anti-inflammatory cytokine IL-10. Additionally, a cohort study by Huang J *et al.* [16] found that maternal PYRs exposure before delivery was positively associated with the incidence of persistent fever and otitis media in 2-year-old children. Animal studies have demonstrated that PYRs exposure can activate the oxidative stress responses [17] and apoptotic signaling pathways [18] in lymphocytes, leading to increased lymphocyte apoptosis. These findings suggest that maternal exposure to PYR during pregnancy may be associated with alterations consistent with DIT and may increase the risk of infections, asthma, and allergic diseases in offspring. However, whether different gestational exposure windows to PYRs produce varying effects on neonatal immune function, and the mechanisms underlying such effects remain unclear.

Neonatal immune function represents a continuation of fetal immune development [10]. Although cord blood cells differ in composition, quantity, and properties from peripheral blood, they reflect hematopoietic changes in the fetus throughout gestation [19]. In this study, we established a birth cohort from the first trimester of pregnancy to validate the impact of maternal PYRs exposure during the whole pregnancy on neonatal immune function and to identify potential sensitive windows for this effect. We also analysed the associations of maternal PYRs metabolite during pregnancy with lymphocyte apoptosis to explore the potential mechanisms underlying the impact of prenatal PYRs exposure on neonatal immune function.

## METHODS

### Study design and population

This birth cohort study investigated the impact of maternal PYRs exposure during pregnancy on neonatal immune function. Women in their first trimester (<13 weeks) were recruited from a hospital in the study area between February 2021 and December 2022. Inclusion criteria for participants were as follows: residency in the urban area for at least one year, naturally conceived singleton pregnancy, non-smokers with no history of alcohol abuse, drug use, or genetic disorders, and voluntary agreement to participate. Exclusion criteria included multiple pregnancies; autoimmune diseases during pregnancy; gestational diabetes requiring insulin therapy; severe liver or kidney diseases; hypertensive disorders of pregnancy; HIV/AIDS; tuberculosis; pregnancy complicated by medical or surgical conditions; use of corticosteroids prior to delivery; and newborns with intrauterine infections, severe asphyxia, or genetic diseases such as primary immunodeficiency disorders. Written informed consent was obtained from all participants. The study was approved by the Medical Ethics Committee of Kunming Medical University (Approval No.: KMMU2021MEC044).

### Questionnaire survey and data collection

Questionnaires were administered during three pregnancy periods: first trimester (6–12 gestational weeks), second trimester (21–24 gestational weeks), and third trimester (33–36 gestational weeks). The questionnaires covered demographic information, living environment, dietary habits, pesticide exposure history, and health status.

Gestational weight gain was categorised as inadequate, adequate, or excessive based on Institute of Medicine guidelines [20]. Passive smoking was defined as exposure to tobacco smoke for ≥15 minutes on at least one day per week. Perceived stress during pregnancy was assessed using the Cohen Perceived Stress Scale (PSS), with an average score ≥14 classified as high perceived stress [21]. Perinatal health information was collected during hospitalisation for delivery. Birth weight z-scores were calculated using age- and sex-standardised growth curves [22]. All medical data were obtained from hospital-registered physicians.

### Urine metabolites of pyrethroid pesticides and creatinine measurements

Spot urine samples (10 mL) were collected as random, midstream voids at the time of routine prenatal care visits, without specific dietary or hydration restrictions, and were stored at −80°C. Urinary PYRs metabolites (3PBA, 4F3PBA, and *cis*-DBCA) were quantified using ultra-high performance liquid chromatography-tandem mass spectrometry (UHPLC-MS/MS; AB SCIEX 3200 Q TRAP, USA) in Yunnan Provincial Key Laboratory of Pharmacology for Natural Products. Quantitative analysis methods were derived from previous established protocols [8,23]. Urinary creatinine levels were measured using the sarcosine oxidase method (Biotek Synergy H1 multifunctional microplate reader, USA) and used to adjust the PYRs metabolite concentrations [24].

### Cord blood lymphocyte subsets cell counts and lymphocyte apoptosis rate measurements

At delivery, 4 ml of umbilical venous blood was collected into EDTA anticoagulant tubes and stored at 4°C. TBNK lymphocyte subset analysis was performed using the BD Multitest 6C-TBNK reagent (BD Biosciences, cat. No. 662967). Data acquisition was performed using a BD FACS Canto II flow cytometer (BD Biosciences) and BD FACS Canto Clinical software (v3.1; BD Biosciences, San Jose, CA, USA). The gating strategy was established as follows:

1. forward scatter height (FSC-H) *vs.* forward scatter area (FSC-A) to exclude cell doublets;

2. live leukocytes identified by FSC *vs.* side scatter (SSC) to eliminate debris and apoptotic bodies;

3. CD45^+^ cells gated to isolate hematopoietic cells;

4. within the CD45^+^ population(lymphocyte), T cells defined as CD3^+^, with subgating into CD4^+^ T cells (CD3^+^CD4^+^) and CD8^+^ T cells (CD3^+^CD8^+^);

5. B cells identified as CD3^-^CD19^+^;

6. NK cells defined as CD3^-^CD16/56^+^.

The absolute count of each lymphocyte subset was calculated using the BD Multitest TBNK Reagent protocol. To ensure analytical consistency, daily quality control was performed using Beckman Coulter whole blood controls, with results monitored *via* Levey-Jennings control charts. Instrument precision was further validated through daily ‘Performance Tracking’ protocols on the flow cytometer, minimising variability caused by inconsistent settings and ensuring data reproducibility across experiments. The calibration was validated by checking the correlation coefficient (r^2^) of the standard curve, which was required to be ≥0.995 for valid experimental runs.

For apoptosis detection, 5 μL of FITC Annexin V (BD Biosciences, cat. No. 556547) and 5 μL of PerCP-conjugated anti-human CD45 antibody (Agilent Technologies, clone HI30 cat. No. 8931017) were added to the 100μL diluted cord blood sample, followed by gentle vortexing and incubated in the dark at room temperature for 15 minutes. After incubation, 5 μL of propidium iodide (PI, included in BD FITC Annexin V Apoptosis Detection Kit I) was added, and cells were incubated for an additional 5 minutes in the dark at room temperature. The staining reaction was stopped by adding 100 μL of 1 × Annexin V Binding Buffer to each tube. Data acquisition was performed within one hour of staining using BD FACS Canto II flow cytometer. Flow cytometry data were analysed using Kaluza software (v2.1; Beckman Coulter, Brea, CA, USA). Lymphocyte apoptosis results were expressed as the percentage of early apoptotic cells relative to total lymphocytes [25].

### Statistical analysis

Statistical analyses were performed using SPSS, version 26 (SPSS, Chicago, IL, USA). Comparisons of urinary PYRs metabolite detection rates and levels across different pregnancy periods were analysed using χ^2^ tests or Kruskal-Wallis H tests. PYRs exposure levels were estimated based on urinary PYRs metabolite concentrations. For metabolite measurements below the limit of detection (LOD), concentrations were imputed as LOD/√2 [26].

Multivariate linear regression models were employed to evaluate associations between maternal PYRs metabolites during pregnancy and neonatal cord blood lymphocyte subsets cell count and lymphocyte apoptosis rates. Given the highly right-skewed distribution of urinary PYR metabolite concentrations in this cohort, with the majority of samples concentrated at low background levels, the creatinine-adjusted concentrations of PYRs metabolites in each trimester were categorised into low- and high-level groups based on the 75th percentile (low-level group: ≤75th percentile, high-level group: >75th percentile), a cutoff that aligns with the public health goal of identifying at-risk subpopulations most likely to benefit from targeted interventions [8]. In addition, based on the number of trimesters with high-level exposure, we categorised the exposure into three groups: high-level exposure in two or more trimesters, high-level exposure in one trimester, and low-level exposure in all three trimesters, and performed the analysis again to assess the cumulative effects of repeated exposure to high levels of PYRs across the three trimesters on neonatal immune function [6].

Covariates were selected through causal inference frameworks, including a DAG developed (Figure S1 in the **Online Supplementary Document**) *via* Dagitty.net [27], with additional adjustments based on established clinical evidence [28]. Dependent variables were log-transformed prior to analysis to mitigate the influence of outliers. Sensitivity analyses were performed using quartile-based categorical exposure and continuous exposure variables. Sensitivity analyses also were conducted by stratifying the study population into vaginal delivery and cesarean section subgroups [29] to assess the robustness of the associations between PYRs exposure and neonatal lymphocyte subsets. Additionally, sex-stratified analyses were performed to examine potential sex-specific differences in these associations. Prior to interpreting the results of the multiple linear regression models used for analysing continuous outcomes, comprehensive residual diagnostics were performed to assess the fulfillment of key statistical assumptions, including linearity (evaluated *via* partial residual plots), normality and homoscedasticity of residuals, and absence of problematic multicollinearity (variance inflation factor, VIF<5). Two-tailed tests were conducted with a significance level of *P*-value <0.05. To address the issue of multiple comparisons, we applied the Benjamini-Hochberg false discovery rate (FDR) correction within each analytical family. FDR-adjusted q-values <0.05 were considered statistically significant after correction; q-values between 0.05 and 0.10 were considered marginally significant.

## RESULTS

### Demographic characteristics

Of the 592 eligible women who provided informed consent, 373 mother-neonate dyads completed all three trimester urine collections, cord blood sampling, and questionnaire assessments and constituted the analytic cohort (**Figure 1**). The mean maternal age at delivery was 30.2 (SD = 3.8) years; 11.6% were aged ≥35 years, and 86.6% had attained more than 12 years of education. Household annual income exceeded 90,000 RMB in 53.4% of cases, while 45.9% exhibited gestational weight gain below recommended thresholds. Passive smoking exposure was reported in 50.1% of mothers, and 60.9% experienced high perceived stress during pregnancy. Among the neonates, 49.1% were male, with a mean gestational age of 39.1 weeks and mean birth weight of 3160 g. Vaginal delivery occurred in 81.0% of cases, with preterm birth observed in 3.5% of neonates. Birth weight z-scores fell within the −1 to +1 range in 73.6% of infants (**Table 1**).

**Figure 1:**
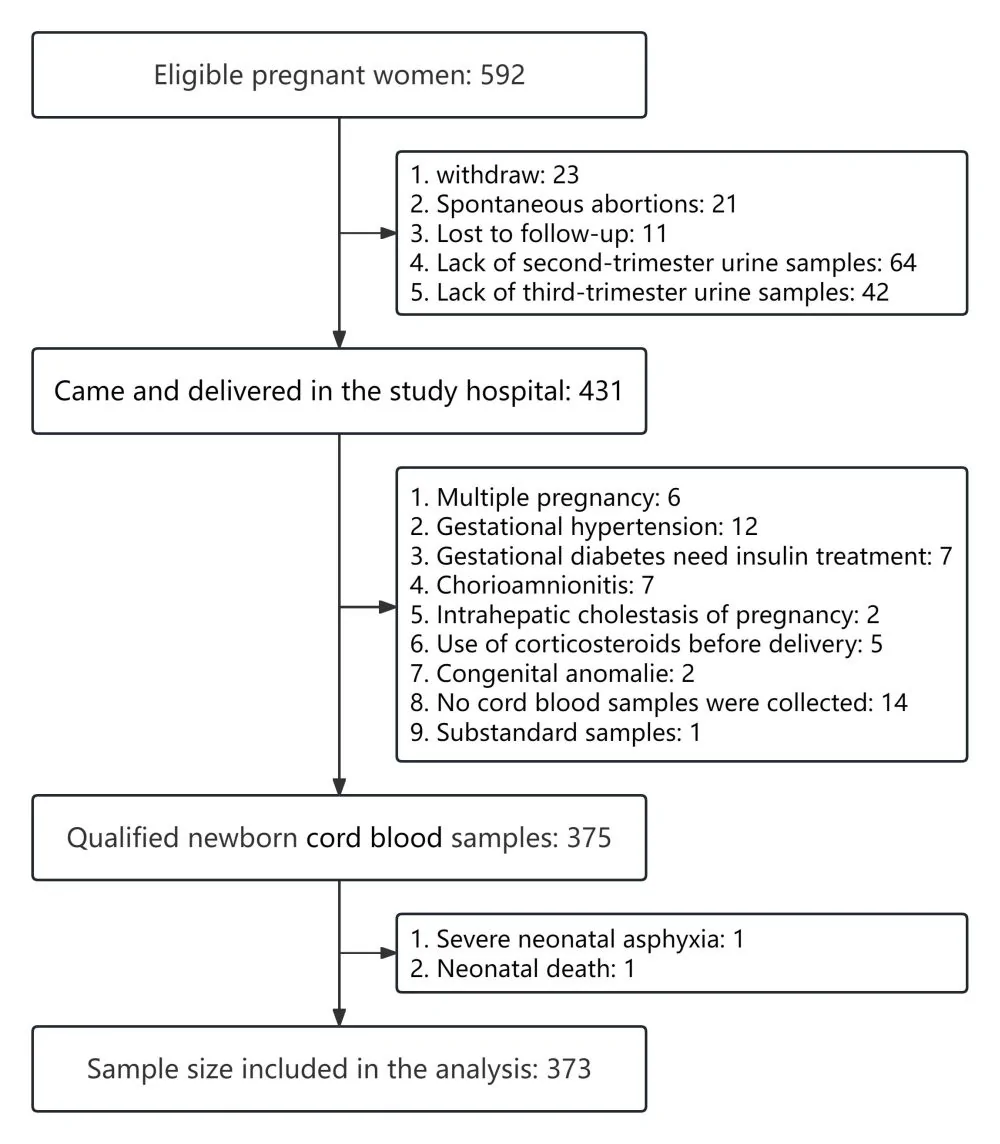
Study flowchart.

**Table 1:** Sociodemographic characteristics of mothers and their children at birth

Characteristics	Mean ± SD or n (%)
**Maternal characteristics**	
Maternal age, in years	
*≤29*	156 (41.8)
*30 ~ 34*	174 (46.6)
*≥35*	43 (11.6)
Educational background	
*12 years and below*	50 (13.4)
*13 ~ 16 years*	269 (72.1)
*Above 16 years*	54 (14.5)
Annual household income (CNY)	
*≤90,000*	174 (46.6)
*>90,000*	199 (53.4)
Gestational weight gain	
*Inadequate*	171 (45.9)
*Adequate*	140 (37.5)
*Excessive*	62 (16.6)
Passive smoking	
*Yes*	187 (50.1)
*No*	186 (49.9)
Perceived stress	
*Low perceived stress*	146 (39.1)
*High perceived stress*	227 (60.9)
**Neonatal characteristics**	
Gender	
*Male*	183 (49.1)
*Female*	190 (50.9)
Gestational age, in weeks	39.1 ± 1.4
Birthweight, in grams	3,160 ± 834
Mode of delivery	
*Vaginal delivery*	302 (81.0)
*Caesarean section*	71 (19.0)
Gestational age (week)	
*<37*	13 (3.5)
*≥37*	360 (96.5)
Birth weight z-score	
*−1 ~ 1*	275 (73.6)
*<−1*	51 (13.7)
*>1*	47 (12.7)

SD – standard deviation

### Concentrations of urine PYRs metabolites

Among maternal urinary PYRs metabolites during pregnancy, 3PBA was detected in 80.4% of samples, with detection rates in the third trimester (67.0%) significantly lower than in the first (χ^2^ = 37.63, *P* < 0.001) and second (χ^2^ = 48.15, *P* < 0.001) trimesters. 4F3PBA was universally detected (100%) across all trimesters. For *cis*-DBCA, the overall detection rate was 80.0%, with third trimester rates (75.1%) lower than those in the first (χ^2^ = 7.30, *P* = 0.007) and second (χ^2^ = 4.95, *P* = 0.026) trimesters (**Table 2**; Table S1 in the **Online Supplementary Document**).

**Table 2:** Maternal urinary concentrations of pyrethroid pesticides metabolites during pregnancy (μg/g, creatinine-adjusted, n = 373)

Exposure	Percentile					Detection	
	10th	25th	50th	75th	90th	n	%
First trimester							
*3PBA*	0.04	0.15	0.36	0.79	1.76	321	86.1
*4F3PBA*	0.19	0.27	0.40	0.66	0.98	373	100.0
*cis-DBCA*	0.11	0.26	0.50	0.88	1.55	310	83.1
Second trimester							
*3PBA*	0.03	0.13	0.31	0.54	0.87	329	88.2
*4F3PBA*	0.17	0.25	0.37	0.55	0.91	373	100.0
*cis-DBCA*	0.07	0.18	0.42	0.78	1.19	305	81.8
Third trimester							
*3PBA*	0.01	0.02	0.11	0.32	0.65	250	67.0
*4F3PBA*	0.15	0.21	0.30	0.46	0.66	373	100.0
*cis-DBCA*	0.08	0.14	0.30	0.62	1.20	280	75.1

3PBA – 3-phenoxybenzoic acid, 4F3PBA – 4-fluoro-3-phenoxybenzoic acid, *cis*-DBCA – cis-2,2-dibromovinyl-2,2-dimethylcyclopropane-1-carboxylic acid

The median detection level of 3PBA was 0.24μg/g (interquartile range, IQR = 0.07, 0.55). Third-trimester 3PBA levels were significantly lower than those in the first (H = 10.69, *P* < 0.001) and second (H = 8.02, *P* < 0.001) trimesters. For 4F3PBA, the median detection level was 0.36 μg/g (IQR = 0.24, 0.54), with third-trimester levels lower than both the first (H = 6.52, *P* < 0.001) and second (H = 4.01, *P* < 0.001) trimesters. The median *cis*-DBCA detection level was 0.41 μg/g (IQR = 0.18, 0.78), with third-trimester concentrations lower than in the first (H = 6.00, *P* < 0.001) and second trimesters (H = 3.17, *P* = 0.002) (**Table 2**).

### Cord blood lymphocyte subset cell counts and lymphocyte apoptosis rates

The median CD3^+^ T cell count was 2284 cells/μL (IQR = 1671, 2932), with CD4^+^ T cells at 1553 cells/μL (IQR = 1124, 2035) and CD8^+^ T cells at 618 cells/μL (IQR = 410, 851). B cell and NK cell counts were 520 cells/μL (IQR = 348, 734) and 658 cells/μL (IQR = 333, 955), respectively (Table S2 in the **Online Supplementary Document**). The mean early apoptosis rate of cord blood lymphocytes was 11.49% (SD = 7.36%) (Table S2 in the **Online Supplementary Document**).

### Associations between prenatal PYRs exposure and lymphocyte subset cell counts

In the third trimester, the high-level group for *cis*-DBCA (>0.78 μg/g, 75th percentile) was negatively associated with cord blood CD3^+^ T cell counts (β = −0.06; 95% CI = −0.12, −0.01, *P* = 0.033; FDR-adjusted q = 0.083) and CD8^+^ T cell counts (β = −0.09; 95% CI = −0.16, −0.01, *P* = 0.028; FDR-adjusted q = 0.083) compared to the low-level group, corresponding to approximately 5.8% and 8.6% reductions in absolute cell counts, respectively (**Figure 2**, Panel A; Table S3 in the **Online Supplementary Document**). Sensitivity analyses using continuous exposure variables and quartile-based categorisation yielded directionally consistent results (Tables S6 and 7 in the **Online Supplementary Document**). The association between high-level group of *cis*-DBCA in the third trimester and cord blood CD3^+^ T cell counts across different delivery modes was statistically consistent with the primary regression analysis (vaginal delivery: β = −0.06; 95% CI = −0.13, 0.01; cesarean section: β = −0.07; 95% CI = −0.19, –0.04). Among neonates delivered vaginally, a significant inverse association was observed between high-level group of *cis*-DBCA in the third trimester and CD8^+^ T cell counts (β = −0.10; 95% CI = −0.19, −0.01). No significant association was found between *cis*-DBCA exposure and CD8^+^ T cell counts in neonates delivered by cesarean section (β = 0.05; 95% CI = −0.20, 0.43).

**Figure 2:**
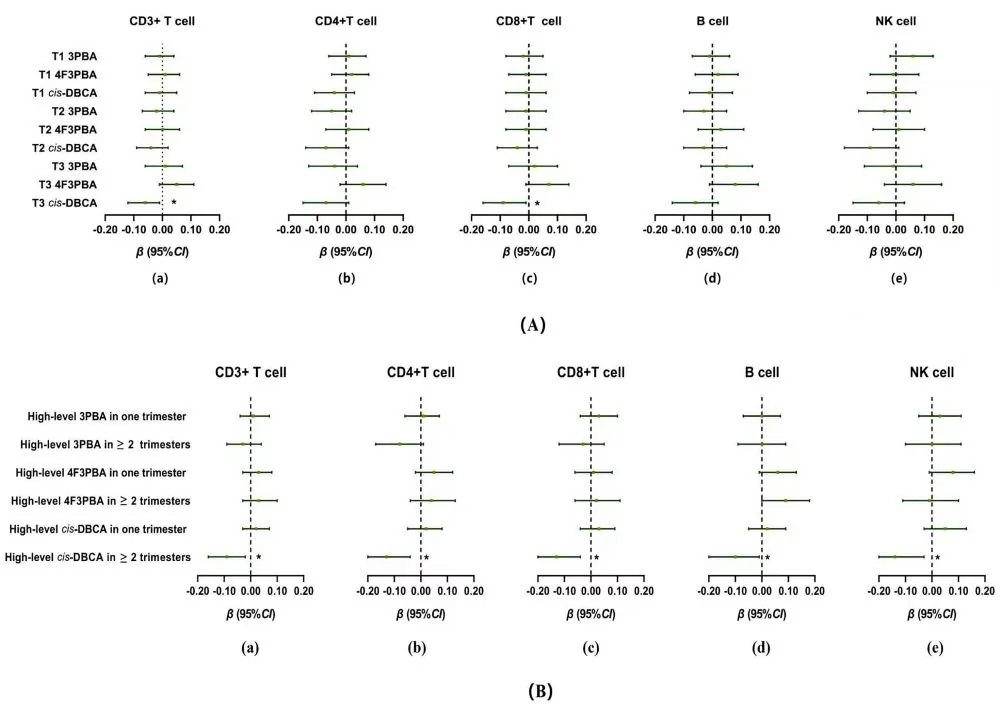
Multiple linear regression for the association between maternal pyrethroid pesticides exposure during pregnancy and neonatal cord blood CD3+ T cell counts (a), CD4+ T cell counts (b), CD8+ T cell counts (c), B cell counts (d) and NK cell counts (e) (β; 95%CI). Panel A. The low-level group was used as the reference. Panel B. The low-level exposure in all three trimesters group was used as the reference. The model was adjusted for maternal age, educational attainment, household income, gestational weight gain, passive smoking during pregnancy, perceived stress during pregnancy, as well as neonatal sex, preterm birth status, delivery mode, and birth weight z-scores. β – beta coefficient, CI – confidence interval, 3PBA – 3-phenoxybenzoic acid, 4F3PBA – 4-fluoro-3-phenoxybenzoic acid, *cis*-DBCA – cis-2,2-dibromovinyl-2,2-dimethylcyclopropane-1-carboxylic acid, T1 – the first trimester, T2 – the second trimester, T3 – the third trimester. *P < 0.05.

Maternal high-level *cis*-DBCA in two or more trimesters was negatively associated with cord blood CD3^+^ T cell counts (β = −0.09; 95% CI = −0.16, −0.02, *P* = 0.012; FDR-adjusted q = 0.019), CD4^+^ T cell counts (β = −0.13; 95% CI = −0.22, −0.04, *P* = 0.006; FDR-adjusted q = 0.015), CD8^+^ T cell counts (β = −0.13; 95% CI = −0.22, −0.04, *P* = 0.006; FDR-adjusted q = 0.015), B cell counts (β = −0.10; 95% CI = −0.20, −0.01, *P* = 0.044; FDR-adjusted q = 0.088), and NK cell counts (β = −0.14; 95% CI = −0.25, −0.03, *P* = 0.015; FDR-adjusted q = 0.019) compared to the low-level *cis*-DBCA group in three trimesters, corresponding to approximately 8.6%, 12.2%, 12.2%, 9.5%, and 13.1% lower absolute cell counts, respectively (**Figure 2**, Panel B; Table S4 in the **Online Supplementary Document**).

### Sex-stratified analyses

In female neonates, high-level *cis*-DBCA exposure in the third trimester was significantly associated with reduced cord blood CD3^+^ T cell counts (β = −0.11; 95% CI = −0.19, −0.03, *P* = 0.009), CD8^+^ T cell counts (β = −0.12; 95% CI = −0.23, −0.02, *P* = 0.022), and NK cell counts (β = −0.13; 95% CI = −0.26, −0.01, *P* = 0.047) compared to the low-level reference group (Table S5 in the **Online Supplementary Document**). In contrast, no significant associations were observed between third-trimester *cis*-DBCA exposure and any lymphocyte subset in male neonates (Table S5 in the **Online Supplementary Document**). Formal tests for the exposure-by-sex interaction terms did not reach statistical significance (all *P* for interaction >0.10).

### Associations between prenatal PYRs exposure and lymphocyte apoptosis rates

In the first trimester, the high-level group for 3PBA was positively associated with cord blood lymphocyte apoptosis rates (β = 0.11; 95% CI = 0.01, 0.22, *P* = 0.038) compared to the low-level group. In the second trimester, the high-exposure group for *cis*-DBCA exhibited higher cord blood lymphocyte apoptosis rates (β = 0.16; 95% CI = 0.04, 0.28, *P* = 0.010) compared to the low-level group (**Table 3**). Maternal repeated high-level *cis*-DBCA in two or more trimesters was positively associated with cord blood lymphocyte apoptosis rates (β = 0.19; 95% CI = 0.03, 0.35, *P* = 0.019) compared to the low-level *cis*-DBCA group in three trimesters (**Table 3**).

**Table 3:** Multiple linear regression for the association between maternal pyrethroid pesticides exposure during pregnancy and neonatal cord blood lymphocyte apoptosis rates (β; 95% CI)

Exposure	Lymphocyte apoptosis rate
Model 1*	
First trimester	
*3PBA*	0.11 (0.01, 0.22)†
*4F3PBA*	−0.01 (−0.13, 0.10)
*cis-DBCA*	0.08 (−0.04, 0.19)
Second trimester	
*3PBA*	0.02 (−0.09, 0.14)
*4F3PBA*	0.04 (−0.08, 0.16)
*cis-DBCA*	0.16 (0.04, 0.28)†
Third trimester	
*3PBA*	−0.04 (−0.18, 0.11)
*4F3PBA*	0.01 (−0.11, 0.14)
*cis-DBCA*	−0.03 (−0.16, 0.10)
Model 2*	
Number of high-level 3PBA in three trimesters	
*1*	0.03 (−0.09, 0.15)
*≥2*	0.11 (−0.04, 0.26)
Number of high-level 4F3PBA in three trimesters	
*1*	0.02 (−0.10, 0.14)
*≥2*	0.04 (−0.11, 0.19)
Number of high-level *cis*-DBCA in three trimesters	
*1*	0.10 (−0.01, 0.21)
*≥2*	0.19 (0.03, 0.35)†

*For Model 1, the low-level group was used as the reference; for Model 2, the low-level exposure in all three trimesters group was used as the reference. The models were adjusted for maternal age, educational attainment, household income, gestational weight gain, passive smoking during pregnancy, perceived stress during pregnancy, as well as neonatal sex, preterm birth status, delivery mode, and birth weight z-scores. β – beta coefficient, CI – confidence interval, 3PBA – 3-phenoxybenzoic acid, 4F3PBA – 4-fluoro-3-phenoxybenzoic acid, *cis*-DBCA – cis-2,2-dibromovinyl-2,2-dimethylcyclopropane-1-carboxylic acid.

†*P* < 0.05.

## DISCUSSION

We established a birth cohort study with enrollment beginning in the first trimester. Our findings indicate that maternal PYRs exposure during pregnancy may reduce neonatal cord blood lymphocyte counts and increase lymphocyte apoptosis rates, thereby adversely affecting neonatal immune development.

Multiple cohort studies have reported detection levels of PYRs metabolites in maternal urine during pregnancy [3]. However, these studies only measured PYRs metabolite levels at a single trimester, which did not reflect the overall exposure throughout the entire gestational period. Our study adopted a repeated sampling strategy with three spot urine samples collected at 3-month intervals, thereby providing a more reliable estimate of PYR exposure throughout pregnancy [30]. Currently, there is limited data on serial measurements of PYRs metabolites during pregnancy. Our previous study reported exposure levels of PYRs metabolites in pregnant women from rural areas [8]. Additionally, a study conducted in Mexico City only measured urinary 3PBA concentrations of 21 subjects during pregnancy [31]. Both studies showed no significant differences in exposure levels across the three trimesters, although we observed that both the detection rates and levels of PYRs exposure decreased as pregnancy progressed. This may be attributed to urban women paying more attention to perinatal care and reducing household pesticides use during pregnancy, as well as pregnancy-related physiological changes in creatinine clearance and urinary dilution [32].

Animal studies have demonstrated that PYRs and their metabolites exhibit strong affinity for CD4, CD8, and CD45 receptors [33], enabling direct binding to immune cells and thereby affecting cellular immune function. These compounds have been shown to induce a significant reduction in CD3^+^, CD4^+^, and CD19^+^ lymphocytes in the spleen of mice in a dose-dependent manner [34]. Population-based studies have also revealed that workers exposed to PYRs exhibit DNA damage in peripheral blood lymphocytes and reduced counts of CD3^+^ and CD4^+^ lymphocytes [35]. Our findings indicate that maternal PYRs exposure during the third trimester leads to decreased cord blood T cell and B cell counts in neonates. Consistent with animal studies, this effect also exhibits dose dependency [34], with significant reductions in neonatal cord blood lymphocyte counts observed only under high-level PYRs exposure (the exposure level of *cis*-DBCA exceeds 0.78 μg/g). Although these associations were only marginally significant after Benjamini-Hochberg FDR correction, they are directionally consistent with our primary results. While they may reflect chance variation due to multiple testing, these exploratory findings provide supportive evidence for a potential adverse effect of prenatal PYRs exposure on neonatal immune development and warrant confirmation in larger cohorts. Notably, sex-stratified analyses revealed that the inverse associations between third-trimester high-level *cis*-DBCA exposure and cord blood lymphocyte cell counts were observed exclusively in female neonates. This pattern may be attributed to the sexually dimorphic pace of fetal thymic maturation [36]. Additionally, PYRs have been demonstrated to possess estrogen-like endocrine-disrupting activity and can interfere with estrogen receptor-α signaling [37], potentially amplifying the disruptive effects on lymphocyte development in female fetuses. However, formal tests for the exposure-by-sex interaction terms did not reach statistical significance, likely reflecting reduced statistical power following sex stratification. Therefore, these sex-specific findings should be interpreted as exploratory and require confirmation in larger cohorts with adequate statistical power to detect effect modification.

The study by Kuper CF *et al.* [10] identifies the human fetal period as critical windows for toxicity. Environmental toxicants may impact the immune system during any of these stages [9]. The impact of PYRs exposure on fetal immune function is related to the development and differentiation of fetal lymphocytes and their sensitivity to environmental toxicants [38]. The hematopoietic inductive microenvironment (HIM) in the bone marrow serves as the origin of immune cells [39]. Fetal bone marrow hematopoiesis begins at approximately 11 weeks of gestation. As hepatic hematopoiesis declines, bone marrow hematopoiesis gradually becomes the primary site of fetal hematopoiesis after 20 ~ 22 weeks of gestation [40]. However, active bone marrow cell proliferation does not commence until 32 weeks of gestation, and hematopoietic stem cells (HSCs) complete their migration from the liver to the bone marrow only by the end of the third trimester [41]. The thymic microenvironment, the site for T cell development, differentiation, and maturation, initiates development around week five of gestation but reaches functional maturity approximately by week 20 [42]. The third trimester represents a pivotal sensitive window for the maturation of lymphocyte cell lineages, as this period is characterised by rapid proliferation and functional differentiation of lymphoid progenitor cells in the fetal thymus, spleen, and bone marrow [43], equivalent exposure levels to environmental toxicants may exert more significant effects on the differentiation and maturation of lymphocytes.

In the current study, no significant effects of PYRs exposure during the first or second trimesters on neonatal cord blood lymphocyte counts were observed, but maternal repeated high exposure to PYRs in two or more trimesters was associated with reduced neonatal lymphocyte counts, and these associations survived FDR correction, suggesting that these effects may persist throughout the entire gestational period and likely result from cumulative impact of maternal repeated high PYRs exposure spanning multiple gestational periods. The third trimester may represent a sensitive window for DIT induced by maternal PYRs exposure. PYRs exposure during the third trimester may disrupt the fetal HIM, affecting bone marrow cell proliferation, particularly the proliferation of HSCs, and thereby impairing neonatal immune function.

Among PYRs, deltamethrin, a type II PYRs, is primarily used for controlling agricultural pests [44] and is the main source of *cis*-DBCA in human biological samples [45]. In our study, only maternal exposure to *cis*-DBCA during pregnancy was found to significantly affect neonatal cord blood lymphocyte counts. This may be attributed to the substantially higher toxicity of *cis*-DBCA to the human body compared to PYRs themselves and their other metabolites [46], or it may be related to the exposure levels of other PYRs metabolites and their differential effects on various aspects of the body [3]. Therefore, reducing the use of type II PYRs in agricultural practices and lowering exposure levels to deltamethrin and its metabolites during pregnancy may help prevent or mitigate the adverse effects of PYRs on immune development.

Research by Kumar *et al.* [33] demonstrated that exposure to deltamethrin in mice induces apoptosis in thymic and splenic cells. This apoptotic response was associated with elevated Caspase-3 mRNA expression levels and subsequent DNA fragmentation, suggesting the involvement of the caspase-dependent intrinsic apoptotic pathway. Permethrin has been found to reduce thymic lymphocyte viability and significantly increase lymphocyte apoptosis [47]. *In vitro* experiments have indicated that cypermethrin induces dose-dependent DNA damage and increases the percentage of apoptotic cells in human lymphocytes [48]. In this study, high level PYRs exposure during pregnancy was associated with increased lymphocyte apoptosis rates in neonatal cord blood, suggesting that maternal PYRs exposure may contribute to DIT in neonates by elevating cord blood lymphocyte apoptosis rates [12]. This effect may represent the cumulative immunotoxic impact of long-term PYRs exposure on lymphocytes during pregnancy [49].

### Strengths and limitations

Our study has several strengths. It is the first birth cohort investigating the impact of maternal PYRs exposure during pregnancy on neonatal immune function. We assessed PYRs exposure levels in daily life across all three trimesters, providing population-based evidence for the DIT associated with maternal PYRs exposure. Several limitations should be acknowledged. We lacked data on unmeasured confounders, particularly co-exposure to other environmental toxicants (*e.g.* organophosphates, heavy metals, air pollutants) [50] that frequently co-occur with PYRs and affect immune development. Consequently, in the absence of multipollutant exposure assessment, we cannot ascertain whether the observed adverse effects on immune development are specific to PYRs exposure or reflects the cumulative impact of the wider prenatal exposome. Future exposome-wide studies measuring correlated toxicants are warranted to clarify this. Our analysis focused solely on neonatal immune function biomarkers measured at birth, which does not fully capture dynamic changes over time. The lack of maternal inflammatory biomarker measurements precludes distinguishing direct fetal immunotoxicity from indirect effects mediated by maternal immune activation [51]. Moreover, without postnatal follow-up data, we cannot assess the potential persistence, evolution, or long-term implications of these effects on immune development beyond the neonatal period.

## CONCLUSIONS

Our study indicates that high maternal PYRs exposure (*cis*-DBCA exceeds 0.78 μg/g) during pregnancy may reduce neonatal cord blood lymphocyte subsets. These effects persist throughout gestation due to cumulative repeated high exposure, with the third trimester potentially representing a sensitive window for adverse effects on immune development. Maternal PYRs exposure may cause DIT by elevating lymphocyte apoptosis rates. Based on these findings, we underscore the critical need for policy interventions to develop alternative pesticides and reduce type II PYRs use, particularly in settings posing high exposure risks to pregnant women. We also recommend incorporating PYRs exposure health education and monitoring into routine prenatal care for high-risk populations.

## Additional material

Online Supplementary Document

## Data Availability

**Data availability:** The data sets used and/or analysed during the current study are available from the corresponding author on reasonable request.
