# Supplementary material for: Impact of prenatal pyrethroid pesticide exposure on neonatal lymphocyte subsets and apoptosis: a birth cohort study
Source: J Glob Health. 2026 Oct 19;16:04318. doi: 10.7189/jogh.16.04318 (PMC13679345; doi:10.7189/jogh.16.04318)
Supplement: Supplementary file 1 [file jogh-16-04318-s001.pdf]

Supplement to: Qi Z, Zhang L, Xiao X, Wu K, Chen S, Song X, Li Y. Impact of prenatal pyrethroid pesticide exposure on neonatal lymphocyte subsets and apoptosis: A birth cohort study. J Glob Health. 2026;16:04318.

Table S1

Maternal urinary concentrations (μg/L) of pyrethroid pesticide metabolites during pregnancy, (N=373).

Exposure	Percentile					Min	Max
	10th	25th	50th	75th	90th		
First Trimester							
3PBA	<LOD	0.19	0.63	1.62	2.94	<LOD	31.12
4F3PBA	0.24	0.36	0.63	1.00	1.47	0.14	6.71
cis-DBCA	<LOD	0.29	0.82	1.66	2.86	<LOD	9.36
Second Trimester							
3PBA	<LOD	0.20	0.51	1.00	1.56	<LOD	12.55
4F3PBA	0.26	0.37	0.57	0.86	1.34	0.14	5.26
cis-DBCA	<LOD	0.18	0.75	1.34	2.24	<LOD	16.77
Third Trimester							
3PBA	<LOD	<LOD	0.20	0.53	1.00	<LOD	11.94
4F3PBA	0.21	0.28	0.42	0.63	1.05	0.14	3.59
cis-DBCA	<LOD	0.10	0.43	1.03	2.00	<LOD	7.96

3PBA – 3-phenoxybenzoic acid, 4F3PBA – 4-fluoro-3-phenoxybenzoic acid, *cis*-DBCA – *cis*-2,2dibromovinyl-2,2-dimethylcyclopropane-1-carboxylic acid; Min – minimum; Max – maximum; LOD – limit of detection.

Table S2

Cord blood lymphocyte subset cell counts and lymphocyte apoptosis rates (N=373).

	$\bar{x} \pm s$	Percentile				
		10th	25th	50th	75th	90th
$CD3^+$ T cell (μL)	2345 ± 961	1092	1671	2284	2930	3573
$CD4^+$ T cell (μL)	1597 ± 683	748	1124	1553	2035	2531
$CD8^+$ T cell (μL)	679 ± 390	281	410	618	851	1138
B cell (μL)	569 ± 314	212	348	520	734	996
NK cell (μL)	717 ± 504	194	333	658	955	1312
Lymphocyte apoptosis rates (%)	11.49 ± 7.36	1.22	4.86	11.24	17.84	21.59

Table S3

Multiple linear regression for the association between maternal pyrethroid pesticides exposure during pregnancy and neonatal cord blood lymphocyte counts [*β* (95%*CI*)].

Exposure	$CD3^+$ T cell	$CD4^+$T cell	$CD8^+$T cell	B cell	NK cell
First Trimester					
3PBA	-0.01(-0.06,0.04)	0.01(-0.06,0.07)	-0.02(-0.08,0.05)	-0.01(-0.07,0.06)	0.06(-0.02,0.13)
4F3PBA	0.01(-0.05,0.06)	0.02(-0.05,0.08)	-0.01(-0.07,0.06)	0.02(-0.06,0.09)	-0.01(-0.09,0.08)
cis-DBCA	-0.01(-0.06,0.05)	-0.04(-0.11,0.03)	-0.01(-0.08,0.06)	-0.01(-0.08,0.07)	-0.01(-0.10,0.07)
Second Trimester					
3PBA	-0.02(-0.07,0.04)	-0.05(-0.12,0.02)	-0.01(-0.08,0.06)	-0.03(-0.10,0.05)	-0.04(-0.13,0.05)
4F3PBA	0.00(-0.06,0.06)	0.01(-0.07,0.08)	-0.01(-0.08,0.06)	0.03(-0.05,0.11)	0.01(-0.08,0.10)
cis-DBCA	-0.04(-0.09,0.02)	-0.07(-0.14,0.01)	-0.04(-0.11,0.03)	-0.03(-0.10,0.05)	-0.09(-0.18,0.01)
Third Trimester					
3PBA	0.01(-0.06,0.07)	-0.04(-0.13,0.04)	0.02(-0.07,0.10)	0.05(-0.04,0.14)	-0.010(-0.11,0.09)
4F3PBA	0.05(-0.01,0.11)	0.06(-0.02,0.14)	0.07(-0.01,0.14)	0.08(-0.01,0.16)	0.06(-0.04,0.16)
cis-DBCA	-0.06(-0.12, -0.01) *	-0.07(-0.15,0.01)	-0.09(-0.16, -0.01) *	-0.06(-0.14,0.02)	-0.06(-0.15,0.03)

The low-level group was used as the reference. The model was adjusted for maternal age, educational attainment, household income, gestational weight gain, passive smoking during pregnancy, perceived stress during pregnancy, as well as neonatal sex, preterm birth status, delivery mode, and birth weight z-scores. β – Beta coefficient; CI – confidence interval; 3PBA – 3-phenoxybenzoic acid, 4F3PBA – 4-fluoro-3-phenoxybenzoic acid, *cis*-DBCA – *cis*-2,2dibromovinyl-2,2-dimethylcyclopropane-1-carboxylic acid. *$P < 0.05$.

Table S4

Multiple linear regression for the association between maternal repeated high exposure to pyrethroid pesticides in three trimesters and neonatal cord blood lymphocyte counts [β (95%*CI*)].

Exposure	$CD3^+$ T cell	$CD4^+$T cell	$CD8^+$T cell	B cell	NK cell
Number of high-level 3PBA in three trimesters					
1	0.01(-0.04,0.07)	0.01(-0.06,0.07)	0.03(-0.04,0.10)	0.00(-0.07,0.07)	0.03(-0.05,0.11)
≥2	-0.03(-0.09,0.04)	-0.08(-0.17,0.01)	-0.03(-0.12,0.05)	0.00(-0.09,0.09)	0.00(-0.10,0.11)
Number of high-level 4F3PBA in three trimesters					
1	0.03(-0.03,0.08)	0.05(-0.02,0.12)	0.01(-0.06,0.08)	0.06(-0.01,0.13)	0.08(-0.01,0.16)
≥2	0.03(-0.03,0.10)	0.04(-0.04,0.13)	0.02(-0.06,0.11)	0.09(-0.01,0.18)	-0.01(-0.11,0.10)
Number of high-level *cis*-DBCA in three trimesters					
1	0.02(-0.03,0.07)	0.02(-0.05,0.08)	0.03(-0.04,0.09)	0.02(-0.05,0.09)	0.05(-0.03,0.13)
≥2	-0.09(-0.16, -0.02) *	-0.13(-0.22, -0.04) *	-0.13(-0.22, -0.04) *	-0.10(-0.20, -0.01) *	-0.14(-0.25, -0.03) *

The low-level group in three trimesters was used as the reference. The model was adjusted for maternal age, educational attainment, household income, gestational weight gain, passive smoking during pregnancy, perceived stress during pregnancy, as well as neonatal sex, preterm birth status, delivery mode, and birth weight z-scores. β – Beta coefficient; CI – confidence interval; 3PBA – 3-phenoxybenzoic acid, 4F3PBA – 4-fluoro-3-phenoxybenzoic acid, *cis*-DBCA – *cis*-2,2dibromovinyl-2,2-dimethylcyclopropane-1- carboxylic acid. *$P < 0.05$.

Table S5

Sex-stratified multiple linear regression for the association between maternal third-trimester pyrethroid pesticides exposure during pregnancy and neonatal cord blood lymphocyte counts [*β* (95%*CI*)].

lymphocyte counts	T3 3PBA		T3 4F3PBA		T3 *cis*-DBCA		*P* for interaction
	Boys	Girls	Boys	Girls	Boys	Girls	
$CD3^+$T cell	0.01(-0.08,0.10)	0.02(-0.08,0.11)	0.05(-0.04,0.14)	0.04(-0.04,0.12)	-0.03(-0.11,0.05)	-0.11(-0.19, -0.03) *	0.150
$CD4^+$T cell	-0.05(-0.17,0.07)	-0.01(-0.13,0.11)	0.06(-0.06,0.18)	0.06(-0.05,0.16)	-0.07(-0.19,0.04)	-0.08(-0.19,0.02)	0.922
$CD8^+$T cell	0.01(-0.11,0.12)	0.04(-0.08,0.17)	0.10(-0.02,0.21)	0.02(-0.08,0.13)	-0.07(-0.18,0.04)	-0.12(-0.23, -0.02) *	0.346
B cell	0.07(-0.06,0.19)	0.03(-0.10,0.17)	0.06(-0.06,0.18)	0.08(-0.03,0.19)	-0.03(-0.15,0.08)	-0.11(-0.22,0.01)	0.301
NK cell	0.01(-0.14,0.15)	-0.01(-0.16,0.14)	0.08(-0.07,0.22)	0.03(-0.10,0.16)	-0.03(-0.17,0.11)	-0.13(-0.26, -0.01) *	0.217

The low-level group was used as the reference. The model was adjusted for maternal age, educational attainment, household income, gestational weight gain, passive smoking during pregnancy, perceived stress during pregnancy, as well as preterm birth status, delivery mode, and birth weight z-scores. β – Beta coefficient; CI – confidence interval; T3 – The third trimester, 3PBA – 3-phenoxybenzoic acid, 4F3PBA – 4-fluoro-3-phenoxybenzoic acid, *cis*-DBCA – *cis*-2,2dibromovinyl-2,2-dimethylcyclopropane-1-carboxylic acid. *$P < 0.05$.

Table S6

Multiple linear regression for the association between maternal pyrethroid pesticide exposure (continuous variable) during pregnancy and neonatal cord blood lymphocyte counts [*β* (95%*CI*)].

Exposure	$CD3^+$ T cell	$CD4^+$T cell	$CD8^+$T cell	B cell	NK cell
First Trimester					
3PBA	-0.01(-0.01,0.01)	-0.01(-0.01,0.01)	-0.01(-0.01,0.01)	0.01(-0.01,0.02)	0.01(-0.01,0.02)
4F3PBA	0.01(-0.05,0.06)	0.01(-0.07,0.08)	0.01(-0.07,0.01)	0.01(-0.07,0.09)	-0.02(-0.11,0.08)
cis-DBCA	0.01(-0.03,0.03)	-0.01(-0.04,0.05)	0.01(-0.03,0.05)	-0.01(-0.05,0.04)	0.01(-0.05,0.05)
Second Trimester					
3PBA	0.01(-0.04,0.04)	-0.01(-0.06,0.04)	0.01(-0.04,0.06)	-0.01(-0.06,0.04)	0.03(-0.03,0.09)
4F3PBA	-0.01(-0.07,0.06)	-0.03(-0.12,0.05)	-0.03(-0.12,0.06)	0.03(-0.06,0.12)	-0.03(-0.14,0.08)
cis-DBCA	-0.02(-0.04,0.01)	-0.01(-0.05,0.02)	-0.01(-0.05,0.02)	-0.01(-0.04,0.03)	-0.01(-0.05,0.04)
Third Trimester					
3PBA	0.01(-0.04,0.04)	-0.03(-0.08,0.03)	0.01(-0.04,0.06)	0.01(-0.05,0.06)	-0.03(-0.09,0.04)
4F3PBA	0.06(-0.01,0.13)	0.07(-0.02,0.16)	0.06(-0.03,0.15)	0.09(-0.01,0.18)	0.10(-0.01,0.21)
cis-DBCA	-0.02(-0.05,0.01)	-0.02(-0.05,0.01)	-0.03(-0.06,0.01)	-0.01(-0.05,0.02)	-0.05(-0.09, -0.01) *

The low-level group was used as the reference. The model was adjusted for maternal age, educational attainment, household income, gestational weight gain, passive smoking during pregnancy, perceived stress during pregnancy, as well as neonatal sex, preterm birth status, delivery mode, and birth weight z-scores. β – Beta coefficient; CI – confidence interval; 3PBA – 3-phenoxybenzoic acid, 4F3PBA – 4-fluoro-3-phenoxybenzoic acid, *cis*-DBCA – *cis*-2,2dibromovinyl-2,2-dimethylcyclopropane-1-carboxylic acid. *$P < 0.05$.

Table S7

Multiple linear regression for the association between maternal pyrethroid pesticide exposure (categorized into tertiles based on the 25th and 75th percentiles) during pregnancy and neonatal cord blood lymphocyte counts [*β* (95%*CI*)].

Exposure	$CD3^+$ T cell	$CD4^+$T cell	$CD8^+$T cell	B cell	NK cell
First Trimester					
3PBA					
Moderate exposure	-0.01(-0.08,0.06)	-0.02(-0.11,0.08)	-0.02(-0.11,0.07)	-0.01(-0.11,0.08)	0.02(-0.09,0.13)
High exposure	-0.01(-0.09,0.06)	-0.01(-0.11,0.09)	-0.04(-0.14,0.06)	-0.01(-0.11,0.10)	0.07(-0.05,0.19)
4F3PBA					
Moderate exposure	-0.03(-0.10,0.03)	-0.03(-0.12,0.05)	-0.02(-0.11,0.06)	-0.02(-0.11,0.07)	-0.02(-0.12,0.09)
High exposure	-0.02(-0.09,0.05)	-0.01(-0.11,0.09)	-0.03(-0.12,0.07)	0.02(-0.09,0.12)	-0.01(-0.13,0.11)
cis-DBCA					
Moderate exposure	-0.03(-0.10,0.04)	-0.03(-0.12,0.06)	-0.01(-0.09,0.08)	-0.13(-0.22, -0.04) *	-0.01(-0.12,0.10)
High exposure	-0.03(-0.10,0.05)	-0.06(-0.16,0.05)	-0.01(-0.11,0.09)	-0.12(-0.22, -0.01) *	-0.03(-0.15,0.10)
Second Trimester					
3PBA					

Moderate exposure	0.03(-0.03,0.10)	0.04(-0.05,0.13)	0.04(-0.05,0.12)	0.07(-0.02,0.16)	0.05(-0.06,0.16)
High exposure	0.01(-0.07,0.09)	-0.01(-0.12,0.09)	0.01(-0.09,0.12)	0.04(-0.07,0.15)	-0.01(-0.14,0.12)
4F3PBA					
Moderate exposure	0.01(-0.05,0.07)	0.02(-0.06,0.09)	0.02(-0.06,0.09)	-0.02(-0.10,0.07)	-0.05(-0.14,0.05)
High exposure	0.01(-0.07,0.08)	0.02(-0.08,0.11)	-0.01(-0.10,0.09)	0.03(-0.07,0.12)	-0.04(-0.15,0.08)
cis-DBCA					
Moderate exposure	0.05(-0.01,0.11)	0.03(-0.04,0.11)	0.07(-0.01,0.14)	0.03(-0.05,0.11)	0.09(-0.01,0.18)
High exposure	-0.01(-0.08,0.07)	-0.05(-0.15,0.04)	-0.01(-0.10,0.09)	-0.02(-0.12,0.08)	-0.03(-0.15,0.08)
Third Trimester					
3PBA					
Moderate exposure	0.03(-0.02,0.08)	0.03(-0.03,0.10)	0.05(-0.02,0.11)	0.05(-0.02,0.12)	0.03(-0.05,0.12)
High exposure	0.03(-0.04,0.10)	-0.02(-0.11,0.07)	0.05(-0.04,0.13)	0.08(-0.02,0.17)	0.01(-0.10,0.12)
4F3PBA					
Moderate exposure	-0.01(-0.06,0.04)	0.01(-0.06,0.071)	-0.01(-0.08,0.06)	0.01(-0.07,0.07)	0.03(-0.06,0.11)

High exposure	0.04(-0.02,0.112)	0.06(-0.03,0.15)	0.06(-0.03,0.15)	0.08(-0.02,0.17)	0.09(-0.02,0.20)
cis-DBCA					
Moderate exposure	0.00(-0.05,0.051)	0.01(-0.07,0.07)	-0.01(-0.07,0.07)	-0.04(-0.11,0.03)	-0.05(-0.14,0.03)
High exposure	-0.06(-0.13,0.01)	-0.07(-0.16,0.02)	-0.09(-0.17,0.01)	-0.07(-0.16,0.02)	-0.09(-0.19,0.02)

The low-level group (<75th percentile) was used as the reference. The model was adjusted for maternal age, educational attainment, household income, gestational weight gain, passive smoking during pregnancy, perceived stress during pregnancy, as well as neonatal sex, preterm birth status, delivery mode, and birth weight z-scores. β – Beta coefficient; CI – confidence interval; 3PBA – 3-phenoxybenzoic acid, 4F3PBA – 4-fluoro-3-phenoxybenzoic acid, *cis*-DBCA – *cis*-2,2dibromovinyl-2,2-dimethylcyclopropane-1-carboxylic acid. *$P < 0.05$.

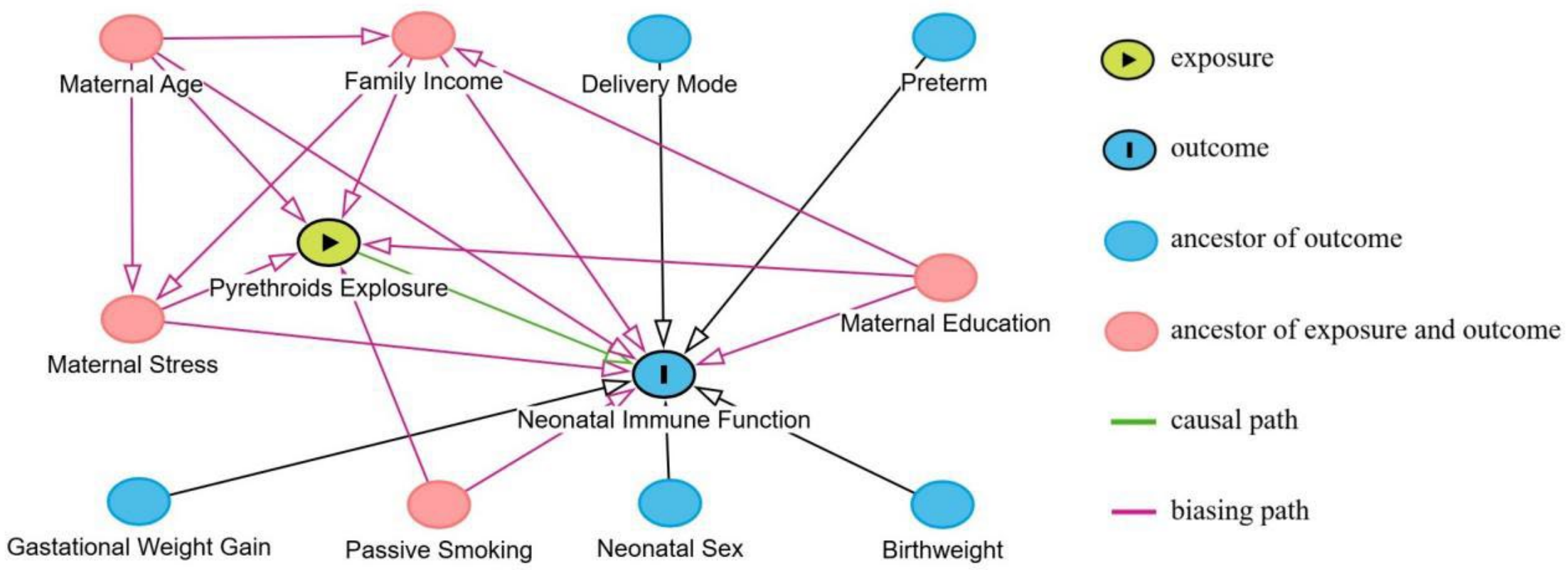

Figure S1. Directed acyclic graph (DAG) for the association between prenatal pyrethroid pesticide exposure and neonatal immune function
